# Publisher Correction: Female behavior drives the formation of distinct social structures in C57BL/6J versus wild-derived outbred mice in field enclosures

**DOI:** 10.1186/s12915-024-01871-8

**Published:** 2024-04-01

**Authors:** Caleb C. Vogt, Matthew N. Zipple, Daniel D. Sprockett, Caitlin H. Miller, Summer X. Hardy, Matthew K. Arthur, Adam M. Greenstein, Melanie S. Colvin, Lucie M. Michel, Andrew H. Moeller, Michael J. Sheehan

**Affiliations:** 1 Laboratory for Animal Social Evolution and Recognition, Department of Neurobiology and Behavior, Cornell University, Ithaca, NY 14853 USA; 2 Department of Ecology and Evolutionary Biology, Cornell University, Ithaca, NY 14853 USA

**Correction: BMC Biology 22, 35 (2024)**

**https://doi.org/10.1186/s12915-024-01809-0**

The production team of the original article [1] mistakenly carried forward the following errors which have since been amended:

• The ORCIDs of all authors post-first author were mistakenly omitted.

• The term, ‘two by four’ was changed to ‘2 × 4’.

• Two instances of the term ‘ad libitum’ were italicised.

• The sentence, ‘The numbers reported here are the average number of hours estimated per trial.’ in the caption of Fig. 4A was changed to ‘The scores reported here are the average number of hours estimated per trial. A white square indicates that a particular combination was not observed.’

• The words ‘Strain and sex’ in the caption of Fig. 4C was replaced by ‘All sex and genotype combinations…’

• The word ‘strains’ in the caption of Fig. 4C was replaced by ‘genotypes’.

• The word ‘outbred’ in the caption of Fig. 4D was replaced by ‘WD’.

• The word ‘strain’ in Fig. 4H was replaced by ‘genotype’.
