# Supplementary figures and images for: Female behavior drives the formation of distinct social structures in C57BL/6J versus wild-derived outbred mice in field enclosures
Source: BMC Biol. 2024 Feb 14;22:35. doi: 10.1186/s12915-024-01809-0 (PMC10865716; doi:10.1186/s12915-024-01809-0)

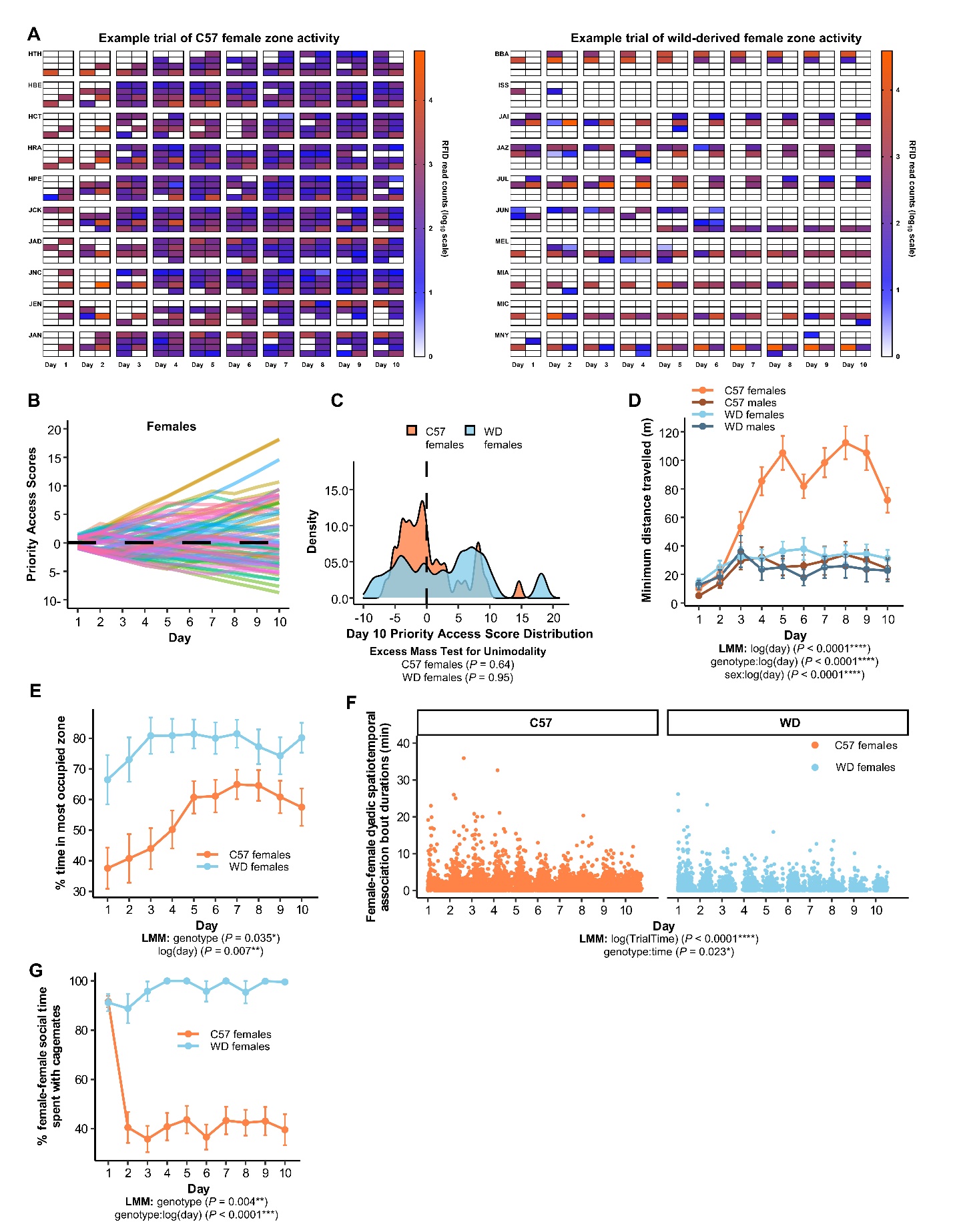

Supplement: Supplementary file 3 — Additional file 3: Figure S3. Distinct patterns of space utilization in C57 females. (A) C57 females extensively explore the available resource zones over the course of the trial compared to WD females. Example full trial data of female zone use in C57 (left) and WD (right) mice. Schematic of the resource zone locations (colored boxes) within the field enclosures (2 x 4 grids) showing patterns of zone usage for animals (rows) across 10 days of activity (columns). White boxes indicate resource zones that were not visited by the focal individual. (B) Daily priority access scores over 10 days of observation for female mice. (C) Distributions of Day 10 priority access scores for female mice are not multi-modal (excess mass test for unimodality from the multimode package), indicating decreased or inconsistent monopolization of resource zones amongst females. Higher scores indicate the extent to which a mouse maintained majority access over one or more resource zones relative to same-sex conspecific competitors (see the “Methods” section for details). (D) C57 female mice (n = 40) differed from C57 male (n = 40) and WD male (n = 29) and female (n = 30) mice in their estimated minimum distance travelled over the course of 10 days. (E) WD females spent more time in their most occupied zone than C57 females. (F) Female-female social grouping bout durations over time. For visualization purposes, the y-axis is cut off at 40 (n = 11,928 events shown out of 11,933 total events). (G) WD females spent nearly all of their female-female social time with cage mates after day 2, in contrast with C57 females who generally spent less than half of their female-female social time with cage mates. Data are plotted as means ± SEM. [file 12915_2024_1809_MOESM3_ESM.docx]
