# Supplementary material for: Female behavior drives the formation of distinct social structures in C57BL/6J versus wild-derived outbred mice in field enclosures
Source: BMC Biol. 2024 Feb 14;22:35. doi: 10.1186/s12915-024-01809-0 (PMC10865716; doi:10.1186/s12915-024-01809-0)
Supplement: Supplementary file 2 [file 12915_2024_1809_MOESM1_ESM.docx]

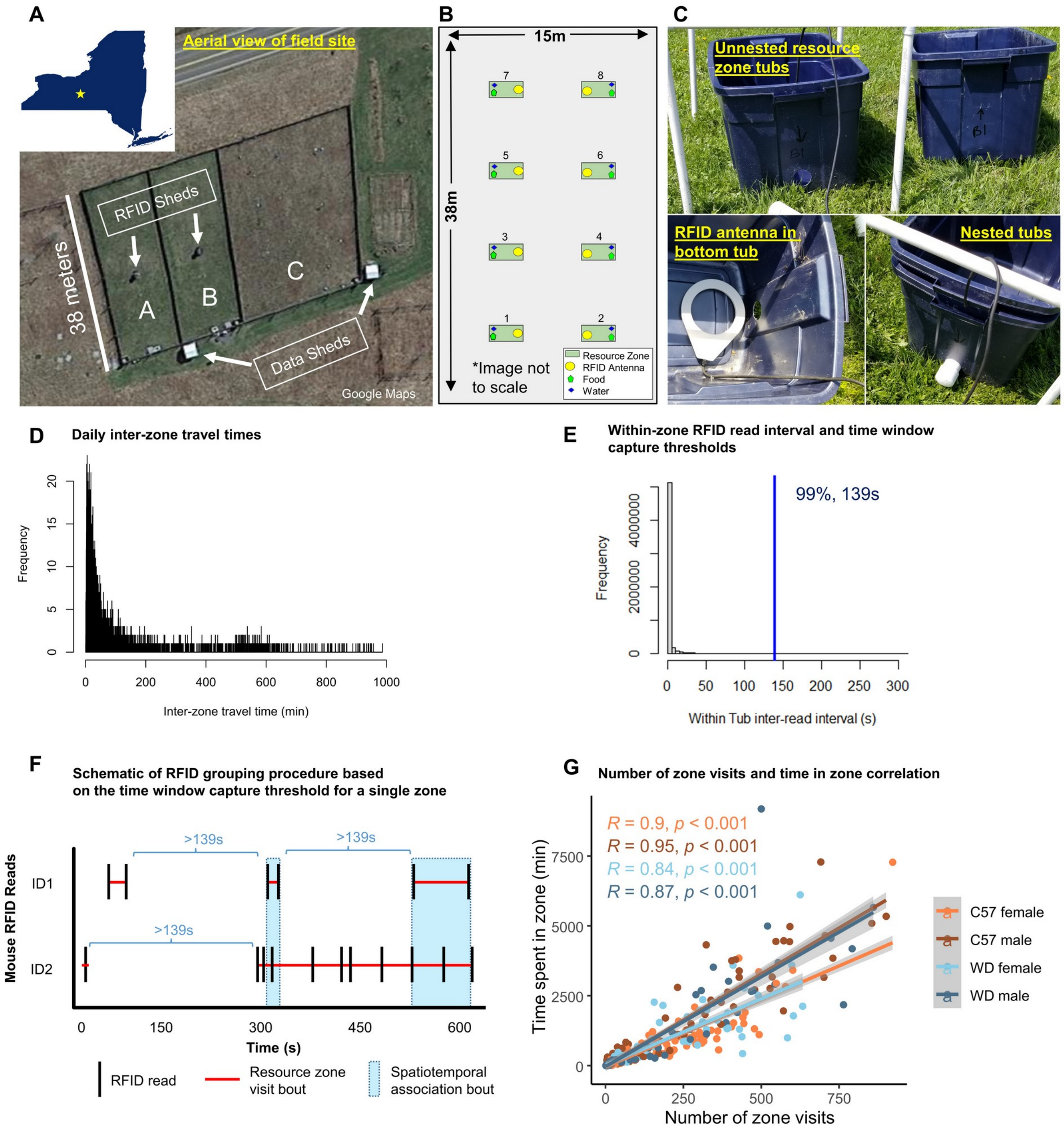
A
Aerial view of field site
RFID Sheds
38 meters
A
B
C
Data Sheds
Google Maps
B
15m
38m
7
8
5
6
3
4
1
2
*Image not to scale
Resource Zone
RFID Antenna
Food
Water
C
Unnested resource zone tubs
RFID antenna in bottom tub
Nested tubs
D
Daily inter-zone travel times
Frequency
Inter-zone travel time (min)
E
Within-zone RFID read interval and time window capture thresholds
99%, 139s
Frequency
Within Tub inter-read interval (s)
F
Schematic of RFID grouping procedure based on the time window capture threshold for a single zone
>139s
Mouse RFID Reads
ID1
ID2
Time (s)
RFID read
Resource zone visit bout
Spatiotemporal association bout
G
Number of zone visits and time in zone correlation
R = 0.9, p < 0.001
R = 0.95, p < 0.001
R = 0.84, p < 0.001
R = 0.87, p < 0.001
Time spent in zone (min)
Number of zone visits
C57 female
C57 male
WD female
WD male
