# Supplementary material for: Female behavior drives the formation of distinct social structures in C57BL/6J versus wild-derived outbred mice in field enclosures
Source: BMC Biol. 2024 Feb 14;22:35. doi: 10.1186/s12915-024-01809-0 (PMC10865716; doi:10.1186/s12915-024-01809-0)
Supplement: Supplementary file 3 [file 12915_2024_1809_MOESM2_ESM.docx]

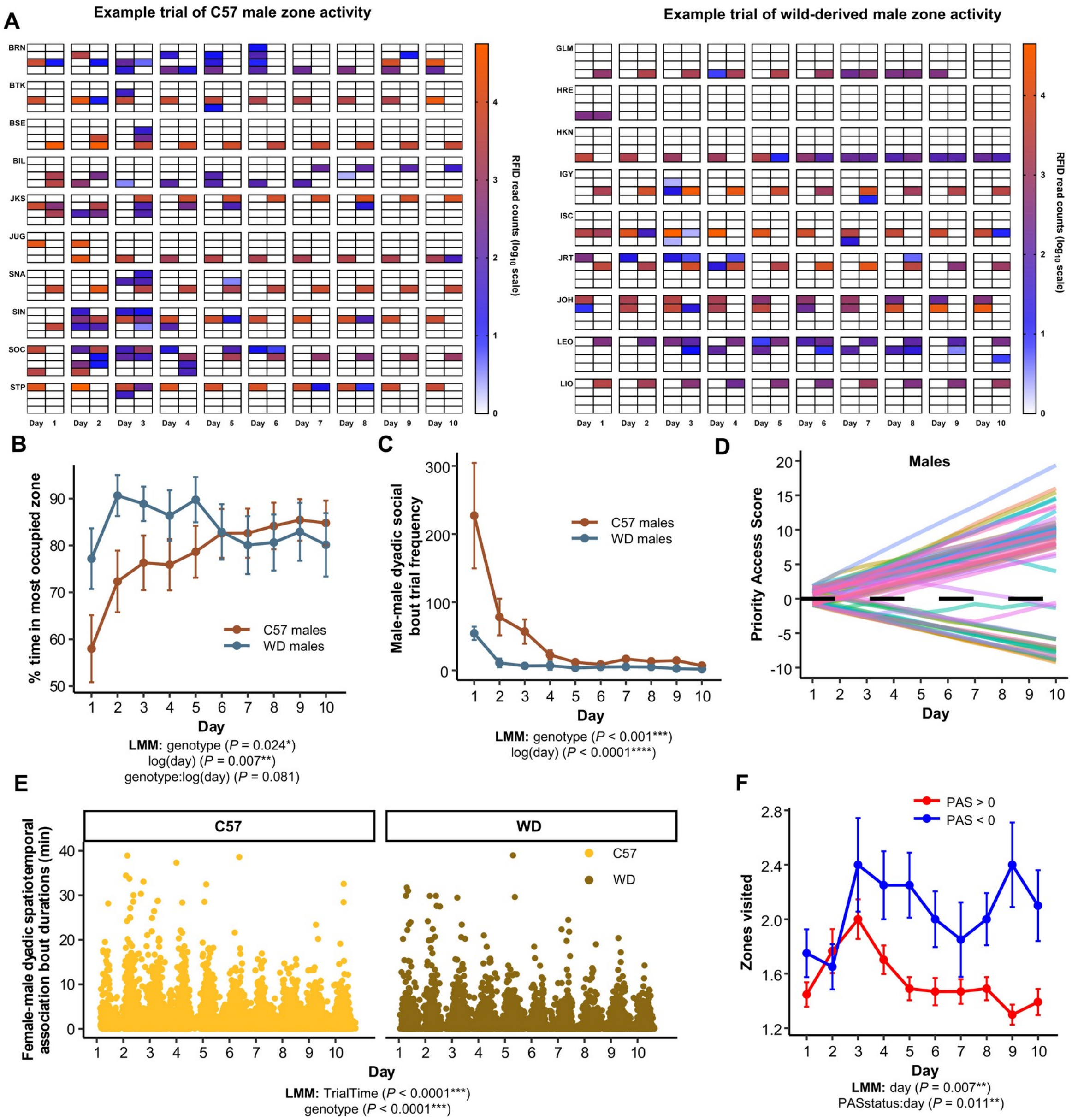
A
Example trial of C57 male zone activity
Example trial of wild-derived male zone activity
BRN
BTK
BSE
BIL
JKS
JUG
SNA
SIN
SOC
STP
GLM
HRE
HKN
IGY
ISC
JRT
JOH
LEO
LIO
RFID read counts (log10 scale)
B
% time in most occupied zone
Day
C57 males
WD males
LMM: genotype (P = 0.024*)
log(day) (P = 0.007**)
genotype:log(day) (P = 0.081)
C
Male-male dyadic social bout trial frequency
Day
C57 males
WD males
LMM: genotype (P < 0.001***)
log(day) (P < 0.0001****)
D
Males
Priority Access Score
Day
E
C57
WD
Female-male dyadic spatiotemporal association bout durations (min)
Day
LMM: TrialTime (P < 0.0001***)
genotype (P < 0.0001***)
F
PAS > 0
PAS < 0
Zones visited
Day
LMM: day (P = 0.007**)
PASstatus:day (P = 0.011**)
