# Supplementary material for: Female behavior drives the formation of distinct social structures in C57BL/6J versus wild-derived outbred mice in field enclosures
Source: BMC Biol. 2024 Feb 14;22:35. doi: 10.1186/s12915-024-01809-0 (PMC10865716; doi:10.1186/s12915-024-01809-0)
Supplement: Supplementary file 4 [file 12915_2024_1809_MOESM4_ESM.docx]

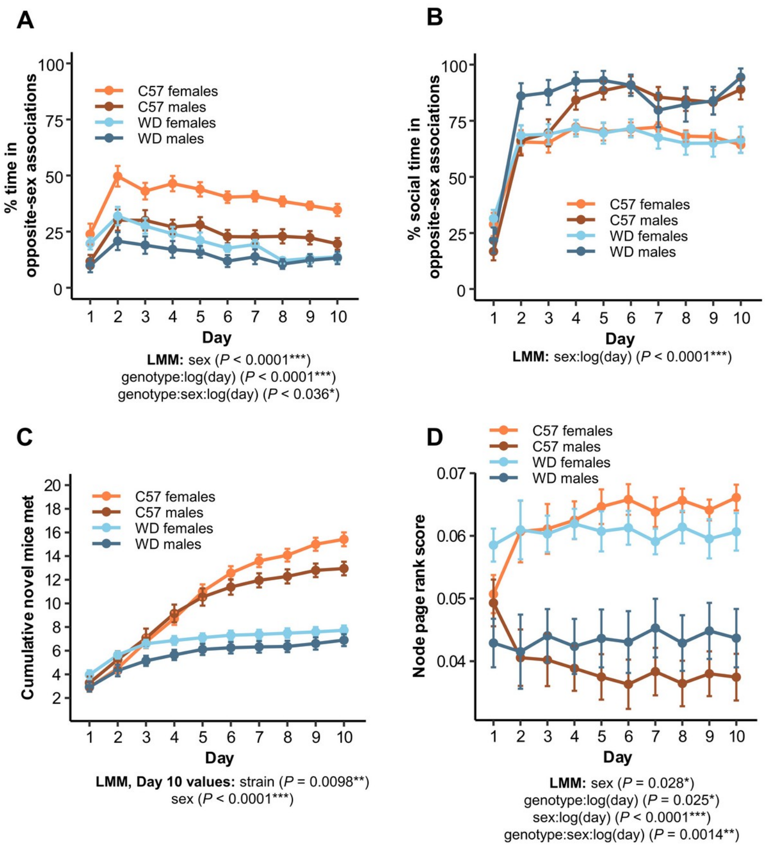
A
% time in opposite-sex associations
C57 females
C57 males
WD females
WD males
Day
LMM: sex (P < 0.0001***)
genotype:log(day) (P < 0.0001***)
genotype:sex:log(day) (P < 0.036*)
B
% social time in opposite-sex associations
C57 females
C57 males
WD females
WD males
Day
LMM: sex:log(day) (P < 0.0001***)
C
Cumulative novel mice met
C57 females
C57 males
WD females
WD males
Day
LMM, Day 10 values: strain (P = 0.0098**)
sex (P < 0.0001***)
D
Node page rank score
C57 females
C57 males
WD females
WD males
Day
LMM: sex (P = 0.028*)
genotype:log(day) (P = 0.025*)
sex:log(day) (P < 0.0001***)
genotype:sex:log(day) (P = 0.0014**)
